# Anatomical Correlates of Non-Verbal Perception in Dementia Patients

**DOI:** 10.3389/fnagi.2016.00207

**Published:** 2016-08-31

**Authors:** Pin-Hsuan Lin, Hsiu-Hui Chen, Nai-Ching Chen, Wen-Neng Chang, Chi-Wei Huang, Ya-Ting Chang, Shih-Wei Hsu, Che-Wei Hsu, Chiung-Chih Chang

**Affiliations:** ^1^Department of Health and Beauty, Shu-Zen Junior College of Medicine and ManagementKaohsiung, Taiwan; ^2^Department of Physical Education, National Kaohsiung University of Applied ScienceKaohsiung, Taiwan; ^3^Department of Neurology, Cognition and Aging Center, Kaohsiung Chang Gung Memorial Hospital, Chang Gung University College of MedicineKaohsiung, Taiwan; ^4^Department of Radiology, Kaohsiung Chang Gung Memorial Hospital, Chang Gung University College of MedicineKaohsiung, Taiwan

**Keywords:** dementia, environmental sound, music, scale-violated melody, semantic dementia

## Abstract

**Purpose**: Patients with dementia who have dissociations in verbal and non-verbal sound processing may offer insights into the anatomic basis for highly related auditory modes.

**Methods**: To determine the neuronal networks on non-verbal perception, 16 patients with Alzheimer’s dementia (AD), 15 with behavior variant fronto-temporal dementia (bv-FTD), 14 with semantic dementia (SD) were evaluated and compared with 15 age-matched controls. Neuropsychological and auditory perceptive tasks were included to test the ability to compare pitch changes, scale-violated melody and for naming and associating with environmental sound. The brain 3D T1 images were acquired and voxel-based morphometry (VBM) was used to compare and correlated the volumetric measures with task scores.

**Results**: The SD group scored the lowest among 3 groups in pitch or scale-violated melody tasks. In the environmental sound test, the SD group also showed impairment in naming and also in associating sound with pictures. The AD and bv-FTD groups, compared with the controls, showed no differences in all tests. VBM with task score correlation showed that atrophy in the right supra-marginal and superior temporal gyri was strongly related to deficits in detecting violated scales, while atrophy in the bilateral anterior temporal poles and left medial temporal structures was related to deficits in environmental sound recognition.

**Conclusions**: Auditory perception of pitch, scale-violated melody or environmental sound reflects anatomical degeneration in dementia patients and the processing of non-verbal sounds are mediated by distinct neural circuits.

## Introduction

Auditory agnosia is a rare disorder characterized by a relatively isolated deficit in auditory comprehension despite normal hearing. Different terms delineate separate entities of auditory agnosia, including word deafness (Ziegler, 1952), environmental sound agnosia (Oppenheimer and Newcombe, 1978) and music agnosia (Ayotte et al., 2002). Lesion studies point to a clear dissociation between the perceptions of different types of sounds, which give hints regarding the anatomical distribution of these unique human abilities (Mendez and Geehan, 1988; Peretz et al., 1994, 1997; Godefroy et al., 1995; Piccirilli et al., 2000; Ayotte et al., 2002; Galaburda et al., 2003; Levitin et al., 2004). Studies on auditory agnosia suggest that the processing of sound is not mediated by a general-purpose auditory architecture but by specialized cortical sub-systems (Thierry et al., 2003). In healthy participants using functional neuroimaging approaches, a dual access route specific for verbal and nonverbal material has been reported, respectively (Thierry and Price, 2006; Hocking and Price, 2009; Gainotti, 2015). Whether these networks also present in patients with degenerative process were not fully examined.

In terms of functional relationships, literature in verbal comprehension emphasizes the importance of the left posterior temporal region (Kreisler et al., 2000). In contrast, melody perception requires an intact auditory cortex and/or posterior superior temporal gyrus on the right hemisphere (Zatorre, 1988; Griffiths et al., 2001; Patterson et al., 2002; Gutschalk et al., 2004). The associated lesions leading to the environmental sound agnosia are not particularly consistent and have included left (Saygin et al., 2003; Vignolo, 2003), right (Griffiths et al., 1997; Mendez, 2001; Vignolo, 2003), bilateral cortical (Matthews et al., 2009), and sub-cortical areas (Tanaka et al., 2002).

Studies on the retention of musical abilities in dementia patients during the degeneration of other cognitive processes provide great insights into the organization of the brain. *Bolero* and the concerto for the left hand composed by Ravel after he had cortico-basal degeneration syndrome represents great musical talent that is innovative and distinct from his previous works (Amaducci et al., 2002). Four patients diagnosed of frontal-temporal dementia (FTD) or semantic dementia (SD) has retained or gained musical skills (Miller et al., 2000) while their loss of left anterior temporal lobes functions was prominent. Studies also indicated a loss of music and non-verbal sound recognition in dementia patients with other diagnosis (Halpern and O’Connor, 2000; Ghacibeh and Heilman, 2003; Matthews et al., 2009). However, no study has explored or compared the perception ability for non-verbal sounds in different types of dementia groups. The investigation of patients having different focal degenerative regions may help to test the hypothesis of conceptual representations of the non-verbal format in the brain (Gainotti, 2015).

Because environmental sound and music perception is so variable, this study aimed to explore the relationships of non-verbal sound perception in different dementia sub-groups and provide anatomical correlations using auditory cognitive tests and voxel-based morphometry (VBM). Based on the review, we hypothesize that dementia patients with greater right temporal atrophy may have worse music perception ability and that environmental sound perception may be related to left, right, or both temporal atrophy. The dementia group with a clinical diagnosis of Alzheimer dementia (AD), behavior variant fronto-temporal dementia (bv-FTD) and SD were selected because they present different degrees of gray matter atrophy in the frontal or temporal lobe. This would allow for group comparisons with controls based on the sound perception scores and elucidation of the anatomic structures related to the task results.

## Materials and Methods

### Subjects, Clinical Evaluation and Cognitive Testing

This study was conducted in accordance with the Declaration of Helsinki and was approved by Chang Gung Memorial Hospital’s Institutional Review Committee on Human Research (97-0255B). The patients and controls were right handed and gave written informed consent to take part. Clinical diagnosis in both patients and controls was determined after a detailed history, neurologic examination, 1-h neuro-psychological battery (Chang et al., 2016a), laboratory screening, and visual inspection of a 3.0-tesla brain magnetic resonance imaging (MRI; Chen et al., 2015). Patients diagnosed with fronto-temporal lobar degeneration met the Neary criteria (Neary et al., 1998). AD was diagnosed according to the International Working Group criteria (McKhann et al., 2011). None of the controls had evidence of impairment during the neuro-psychological testing nor did they have any history of neurologic or psychiatric disorder (Huang et al., 2015). Two sub-groups of fronto-temporal lobar degeneration, the behavior variant, also called bv-FTD, and the temporal variant, also called SD, were included.

### Experimental Design and Materials

#### Neuropsychological Tests

All of the participants were tested for hearing ability using brainstem auditory evoked potentials with the measurement of waves I–V peak latency and waves I–III inter-peak latency. All had corrected-to-normal vision. Participants with a prolonged wave I peak latency (>1.8 ms) were excluded (Burdo, 1989). General intellectual function was assessed using the Mini-Mental State Examination (Folstein et al., 1975). Verbal and non-verbal episodic memory was assessed using a modified California Verbal Learning Test-Mental Status (Chang et al., 2010) and the Rey-Osterrieth Complex Figure Test after a 10-min delay (Boone, 2000). The language screening included the 15-item Boston Naming test (Kaplan et al., 1983) and semantic verbal fluency tests. The subjects’ visual-spatial abilities were assessed by a modified Rey-Osterrieth Complex Figure Test and the number-location test from the Visual Object and Space Perception Battery (Warrington and James, 1991). In addition, the frontal lobe function was assessed using the digit-backwards.

#### Auditory Perceptual Tasks

We used three auditory perceptual tasks to determine the pitch, melody and environmental sound perception. The tasks were performed using a desktop computer connected to two speakers. Before the test, the examiners ensured that the volume of the speakers was sufficiently audible. Following oral instructions and trials, responses were scored on answer sheets. Repetition of the stimuli was allowed once for the environmental sound task if the participant failed to respond to the sound.

#### Pitch (Tasks 1) and Melody Perception Tests (Task 2)

The pitch and melody perception tests required the participants to determine whether two presenting pitches or melodies were identical or not. Instructions were given orally as follows: “I am going to play two notes (melodies). I would like you to tell me if they are the same or different. I will start by playing a sample for you.” A target tone (melody) was followed by a comparison tone (melody) that was the same or different. Participants were given two examples to practice. The examiner ensured that they understood the task and could answer either “same” or “different” after the stimulus.

For the pitch discrimination test, twenty paired tones were played. The pitches were first recorded into a musical instrument digital interface file and then converted to a wave file, using a grand piano as the playback instrument and a sound module (Roland Canvas SC-8850) as the sound source. The pitch interval between the two different tones varied from a minimum of 5.96% and a maximum of 25.9%. The time gap between two notes was 1 s. The frequency of each tone was as follows: C1 = 261.6 Hz; C#1 = 277.2 Hz; D1 = 293.7 Hz; Eb1 = 311.1 Hz; E1 = 329.6 Hz; F1 = 349.2 Hz; F#1 = 370.0 Hz; G1 = 392.0 Hz; Ab1 = 415.3 Hz; A1 = 440.0 Hz; Bb1 = 466.2 Hz; and B1 = 493.9 Hz. For the 20 paired notes, 10 were the same and the other 10 were different. They were mixed randomly to avoid analysis errors.

For the melody perception test, the scale discrimination test of the Montreal Battery of Evaluation of Amusia (MBEA) was used (Peretz et al., 2003). It was a “same-different” discrimination test with manipulation made to a comparison melody that consisted of a scale-violated tune. The pitch was modified to be out of scale, while retaining the original melodic contour; the change was salient because the modified pitch sound was out of tune. The duration of each melody lasted from 3.8 to 6.4 s [mean, 5.1 s; Peretz et al., 2003]. Subjects were required to use a “same-different” classification: on each trial, they had to judge whether the target and the comparison sequence were the same or not. After two practice trials, 31 paired melodies were presented, with each new stimulus preceded by a warning beep. The average time to perform the whole battery was 12 min.

#### Environmental Sound Naming and Matching Test (Task 3)

**Figure 1:**
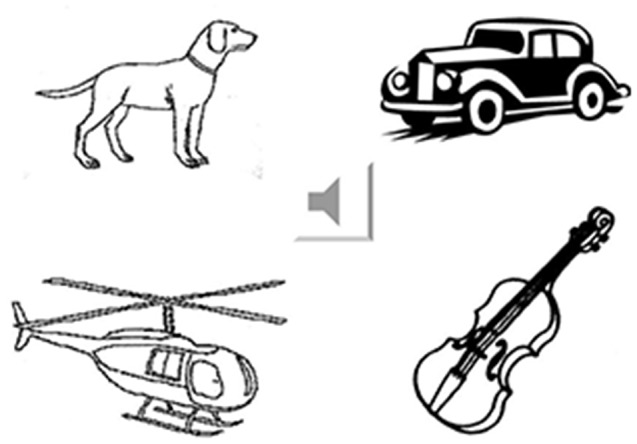
**Example of the environmental sound naming and matching test.** The sound icon was connected to the wave file that played the sound of a car horn. The four pictures represent irrelevant living item (a dog), answer (car), category-related item (helicopter) and supra-ordinate non-living item (violin).

Fifty-five environmental sounds were selected as the sound stimuli (Marcell et al., 2000). These were divided into four categories: animal (*n* = 13), human (*n* = 15), musical instruments (*n* = 12), and environmental noise (*n* = 15). For each category, the sounds were matched by familiarity and complexity based on normative data (Marcell et al., 2000). First, the participants were instructed to listen to the sound and verbally answer what it represented (Marcell et al., 2000). After each response, four pictures appeared on the computer screen (Figure 1). The participants were instructed to point to the most appropriate picture corresponding to the sound even if they could name the sound. The pictures were mostly selected from the Snodgrass and Vanderwart (1980) set. Pictures not present in this set were drawn manually using the same black and white sketch.

All of the pictures represented unique scenes corresponding to unique sounds. Ninety-four different pictures (with different picture complexity and familiarity scores) appeared randomly in various combinations. On average, each picture appeared as a stimulus or distracter 2–3 times. To further categorize the sound stimuli, the pictures were grouped in four categories using the following criteria: (1) correct natural source of the sound (car horn); (2) natural sound source within the same category of stimulus (helicopter); (3) natural sound within the living vs. non-living supra-ordinate category (musical instrument: violin); and (4) irrelevant in any category (dog). Task 3 required 30 min to complete.

### MRI Acquisition

Brain MRI was obtained using a 3.0-T scanner (Excite^®^, GE Medical System, Milwaukee, WI, USA) equipped with echo-planar capability. The T1 inversion recovery prepared three-dimensional spoiled gradient-recalled acquisition in steady state sequence, which were prepared with the following parameters: TR 8.6, TE minimal, Prep time 400, FOV 24, slice thickness 1.5 mm, bandwidth 25, flip angle 15, 128 slabs, 256 × 256 matrix, 1 NEX, phase FOV 1, frequency direction A/P for axial sections.

### Statistical Analysis

#### Neuropsychological and Perceptual Tasks

Statistical analysis explored differences among the groups in terms of demographic data, neuro-psychological performances, and three non-verbal perceptual tests. Statistical analysis was carried out using the Statistical Package for Social Sciences (SPSS) software package (version 10.05 for Windows^®^, SPSS Inc, Chicago, IL, USA). Because the samples were small and were not in a normal distribution, non-parametric tests were applied for comparison between sub-groups. Kruksal-Wallis test was used for overall group statistics while the Mann-Whitney test was used between two group comparisons. Pearson’s correlation or partial correlation was used to calculate the strength of the relationship between continuous variables. A *p* < 0.05 was considered statistically significant.

#### VBM

The VBM protocol was carried out using the Statistical Parametric Mapping 8 package^1^ and standard procedures. Normalized, segmented, and modulated gray matter images were spatially smoothened with a 12-mm full width at half-maximum isotropic Gaussian kernel. Age, gender, and MMSE were regarded as nuisance variables. The global level of atrophy was assessed by measuring the total intra-cranial volume in each image. The significance of each effect of interest was determined using the theory of Gaussian fields. A statistical threshold of *p* < 0.05 corrected for multiple comparisons was accepted.

Scores from the pitch perception test were not included in the analysis of VBM because of variable frequencies between each pitch interval.

For melody perception analysis, patients who scored below the cut-off point of 23 (Ayotte et al., 2002) in the MBEA scale-violated test were first selected as one group and compared with the controls to obtain the region of interest. In addition, morphometric data from the significant region of interest were extracted and correlated with task scores. A linear regression model was constructed for functional interpretation.

For the environmental sound test, a covariant-only statistical model was used to correlate picture-matching scores and gray matter volumes. All of the patients were merged into a single group, regardless of clinical diagnosis.

## Results

### Demographic Data

The 60 subjects were divided into four groups: 16 AD patients, 15 bv-FTD, 14 SD, and 15 controls. Of the SD group, nine were categorized as left temporal variants (SD-L) and five were right temporal variants (SD-R) based on the asymmetry of temporal lobe atrophy (Thompson et al., 2003). There were no between-group differences in age and education (Table 1). The three dementia groups had a significantly lower mini-mental status examination (MMSE) and clinical dementia rating (CDR) scores compared to the controls. The results of other neuro-psychological evaluations were summarized in Table 1.

**Table 1:** **Demographic data of participants in the study**.

Demographic data	Control	AD	bv-FTD	SD
Gender (male:female)	7:8	7:9	9:6	6:8
Age (years)	65.3 (5.9)	63.1 (6.4)	62.1 (6.7)	64.3 (6.4)
mini-mental status examination (30)	28 (0.3)	21.7 (5.5)*	23.3 (4.8)*	21.2 (4.9)*
Clinical dementia rating score	0	0.5–2 (0.83)*	0.5–2 (1.0)*	0.5–2 (0.78)*
Educational level (years)	12.1 (2.3)	10.5 (3.1)	11.4 (2.7)	10.6 (3.4)
Verbal memory 10 min recalls (9)	7.2 (1.1)	3.6 (2.5)*	3.5 (3.1)*	1.3 (2.3)*
Rey- Osterrieth figure copy (17)	16.3 (1)	13 (5.5)	15.2 (2)	15.2 (1.8)
Rey-Osterrieth complex 10-min recall (17)	12.7 (3.1)	6.9 (6.7)*	7.3 (5.5)*	6.5 (5.8)*
Semantic fluency (animal)	23.2 (4.2)	11.5 (8.6)*	12.2 (6.8)*	3.5 (2.7)*^††^
Semantic fluency (fruit)	17.2 (4.5)	10.9 (5.3)	9.4 (5.6)*	4.3 (2.3)*^†^
Digit backward	5.8 (1.4)	3.9 (1.6)*	4.5 (1.2)	4.5 (1.1)
Visual object and space perception battery (10)	9.3 (0.9)	6.5 (3.5)*^§^	8.9 (1.1)	9.6 (0.5)
Boston naming test (15)	14.2 (0.6)	13.4 (3.2)	11.4 (3.1)*	2.4 (2.5)*^§§^

Data represent means and standard deviation in parentheses. Numbers in parentheses following the tasks name indicate the maximal score. AD, Alzheimer dementia; bv-FTD, behavior variant fronto-temporal dementia; SD, semantic dementia; MMSE, mini-mental status examination; CDR, Clinical dementia rating; *P < 0.05 compared with control; ^††^P < 0.05 compared with AD and FTD; ^†^P < 0.05 compared with AD; ^§^P < 0.05 compared with SD; ^§§^P < 0.001 compared with AD.

### Pitch Perception

**Figure 2:**
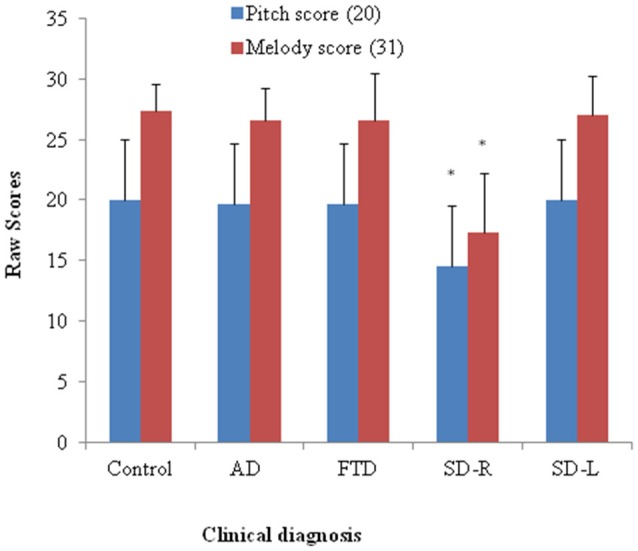
Bar graph with *y*-axis indicating mean and error bar as standard deviation of the pitch (maximal score = 20) or melody scores (maximal score = 31) in the dementia and control groups; *p < 0.01. AD: Alzheimer’s disease; FTD: behavior variant fronto-temporal dementia; SD-R: right temporal variant sementic dementia; SD-L: left temporal variant semantic dementia.

Patients with SD scored lowest among four groups (*χ*^2^ = 11.35, *df* = 3, Kruksal-Wallis test, *p* = 0.01) while the AD, bv-FTD, and control groups were not different. Inspection of the mean scores showed that scores of SD-R contributed to the differences (Figure 2). Further analysis between SD-R and controls showed a significant difference (*χ*^2^ = 9.71, Mann-Whitney test, *p* < 0.01) while SD-L was not significant (*χ*^2^ = 2.23, Mann-Whitney test, *p* > 0.05). Among the dementia patients, the pitch score inversely correlated with the CDR (*r*= −0.336, *p* = 0.042) but not the MMSE score (*r* = 0.285, *p* = 0.087). On the account of near-ceiling performance on the task, we did not perform the correlation analysis.

### Melody Perception

The SD group had the lowest overall ranking among the groups in melody perception test (*χ*^2^ = 16.27, *df* = 3, Kruksal-Wallis test, *p* = 0.001). The SD-R but not the SD-L showed a significant difference with controls (*χ*^2^ = 10.1, Mann-Whitney test, *p* < 0.01, Figure 2). The AD, bv-FTD, and control groups were not different. There was a strong positive partial correlation between pitch and melody scores (*r* = 0.53, *p* = 0.0001) when controlling for the MMSE and CDR scores.

### Environmental Sound Naming and Matching Test

#### Spontaneous Naming

**Figure 3:**
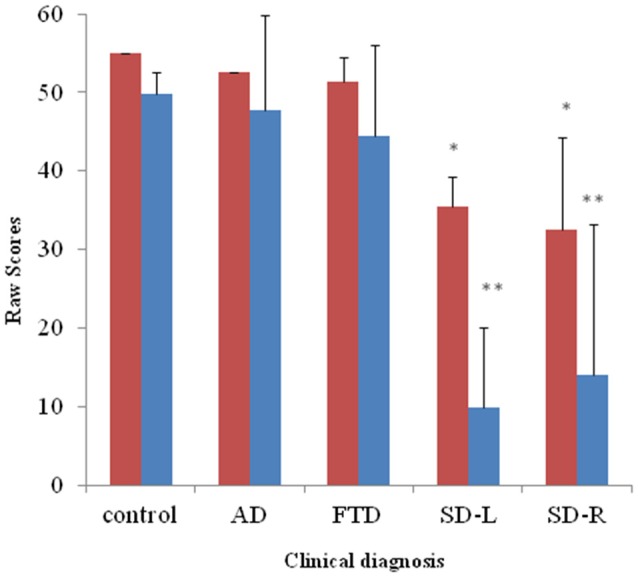
Bar graph with *y*-axis indicating mean and error bar as standard deviation of environmental sound naming (blue; maximal score = 55) and picture matching scores (red; maximal score = 55) in the dementia and control groups; *p < 0.01, **p < 0.001. AD: Alzheimer’s disease; FTD: behavior variant fronto-temporal dementia; SD-R: right temporal variant sementic dementia; SD-L: left temporal variant semantic dementia.

For the spontaneous naming of environmental sounds, the SD group was the only group that showed group differences (*χ*^2^ = 25.085, *df* = 3, Kruksal-Wallis test, *p* < 0.001, Table 2, Figure 3). Total scores in environmental sound naming did not correlate with pitch scores (*r* = −0.36, *p* = 0.40) or melody scores (*r* = 0.02, *p* = 0.458) when controlling for the CDR and MMSE scores. There was no category specificity in naming scores, whether comparing the four categories or the living vs. non-living categories among the dementia groups.

**Table 2:** **Individual scores in the environmental sound test**.

	AD	bv-FTD	SD-L	SD-R	Control
**Sound naming**	47.67 (12.2)	44.38 (11.7)	9.78 (10.3)*	14.00 (19.3)*	49.80 (2.8)
Animal (13)	12.00 (2.7)	10.25 (3.4)	1.89 (2.1)*	1.0 (1.4)*	10.9 (1.9)
Environmental sound (15)	12.67 (3.6)	12.3 (2.4)	2.44 (3.2)*	4.33 (5.9)*	13.5 (1.1)
Music instrument (12)	10.78 (4.7)	10.38 (4.1)	0.78 (0.83)*	2.75 (5.5)*	11.0 (0.9)
Human (15)	12.22 (2.6)	11.5 (4.0)	4.67 (5.5)*	4.67 (5.7)*	14.4 (0.8)
**Picture matching**	52.56 (3.1)	51.38 (3.8)	35.44 (11.8)*	32.50 (17.1)*	55.00 (0)
Animal (13)	12.67 (0.7)	12.13 (1.4)	6.67 (4.5)*	5.75 (5.1)*	13 (0)
Environmental sound (15)	14.33 (1.0)	14.00 (1.1)	12.0 (2.9)*	10 (4.4)*	15.0 (0)
Music instrument (12)	11.2 (1.3)	11.1 (1.0)	6.0 (3.0)*	6.75 (4.5)*	12.0 (0)
Human (15)	14.33 (1.1)	14.13 (1.1)	10.78 (3.0)*	10 (3.6)*	15.0 (0)

Numbers in parentheses following the task names indicate the number of sounds in each category. Data represent means (standard deviation). AD, Alzheimer’s dementia; bv-FTD, behavior variant fronto-temporal dementia; SD-L, left temporal variant of semantic dementia; SD-R, right temporal variant of semantic dementia. *p < 0.001 compared with controls, AD and bv-FTD.

#### Sound-picture Matching

For sound-picture matching, the SD group ranked lowest among the four groups (*χ*^2^ = 26.66, *df* = 3, Kruksal-Wallis test, *p* < 0.001, Table 2, Figure 3) while the AD, bv-FTD, and control groups were not different. Scores in the sound-picture matching correlated positively with sound naming (*r* = 0.88, *p* = 0.001) and MMSE scores (*r* = 0.293, *p* = 0.04), but not with CDR (*r*= −0.055, *p* = 0.38), pitch (*r*= −0.68, *p* = 0.349) or melody scores (*r* = 0.1, *p* = 0.29). In each group, there was no difference in scores for the living vs. non-living supra-ordinate category or in scores for all of the four sub-categories. The SD-L group was more apt at sound matching than the SD-R group (Figure 3).

In error analysis, all of the patients made more errors by subcategorizing pictures as “same” for the living vs. non-living category rather than as “supra-ordinate” or “irrelevant.” For the SD patients, the percentage of errors were 63.4% (same), 25.2% (supra-ordinate) and 1.14% (irrelevant).

### VBM with Functional Correlation

#### Melody Perception Test

**Figure 4:**
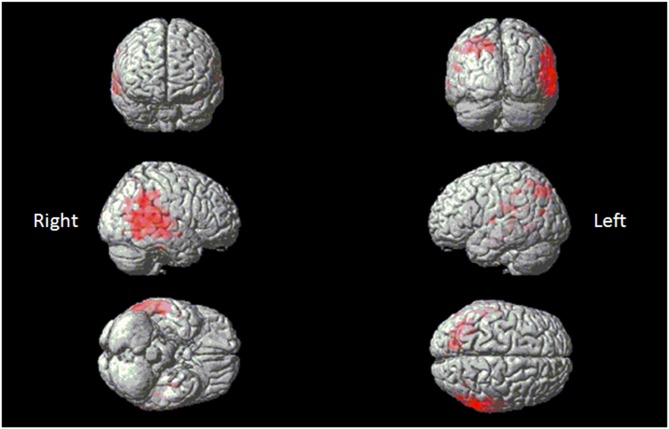
**Red clusters indicate a comparison in the poor melody group (*n* = 11) in dementia patients with control group overlaid on the Statistical Parametric Mapping template.** It shows regions of the right supra-marginal and angular, superior temporal and middle temporal areas.

Eleven patients scored below the cut-off point of 23 on the MBEA scale violated scores: four with AD, one bv-FTD, one SD-L and five SD-R. A voxel-wise comparison of the gray matter between the poor melody score group and the controls showed a significant gray matter loss in the right supra-marginal and angular gyri (Brodmann’s area 39 and 40), as well as in the posterior third of the right superior and middle temporal gyri (Figure 4; Table 3).

**Table 3:** **Result of the Voxel-based Morphometry (VBM) analysis**.

Brain region (BA)	*x*	*y*	*z*	*t* value	*z* score
**Poor melody discrimination effect**	
R supramarginal gyrus (BA 40)	63	−48	38	7.86	4.88
R superior temporal gyrus	60	−34	16	7.72	4.84
R primary and association auditory cortex	66	−18	10	7.26	4.68
(BA 41 and 42)	44	−32	6	7.45	4.75
R middle temporal gyrus	49	−78	−8	6.52	4.42
**Impaired sound–picture-matching effect**	
L hippocampus	−30	−14	−18	7.27	4.84
L mid temporal pole	−36	24	−36	7.06	4.76
L amygdala	−30	−2	−20	6.41	4.51
L superior temporal pole	−34	24	−30	5.92	4.31
R parahippocampus	28	6	−30	5.84	4.27

*R, right; L, left; BA, Brodmann’s area. Threshold of significance is p < 0.05 corrected for multiple comparisons*.

**Figure 5:**
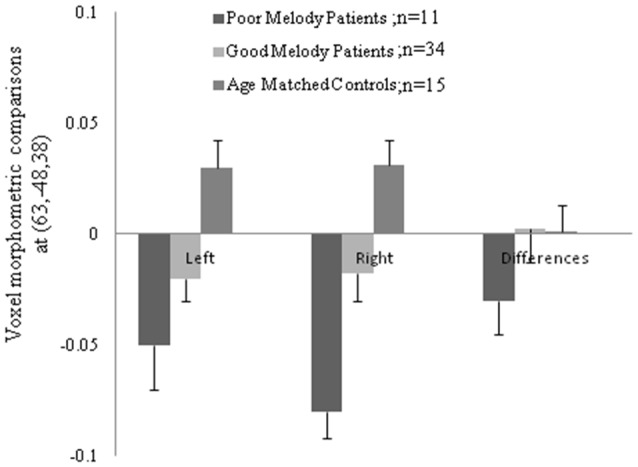
**Morphologic data from both supra-marginal gyri in three groups.** Difference (right minus left) and the numbers in the parenthesis indicated the Montreal Neurological Institute coordinate located within the supra-marginal gyrus.

Meanwhile, morphometric data extracted from the supra-marginal gyrus were shown in Figure 5. The right supra-marginal gyrus in the poor melody score group (*n* = 11) was significantly atrophic compared to the patient group with scored above the cut-off values (*n* = 34) or with controls. Further, the correlation between melody scores and morphometric data by VBM showed that the right supra-marginal, superior and middle temporal region, and primary and secondary auditory cortex volumes were inversely correlated with melody scores (*p* < 0.05). However, only the right supra-marginal and superior temporal regions fitted the linear regression model (*Rsq* = 0.203, *p* = 0.005).

#### Environmental Sound Naming and Matching Test

**Figure 6:**
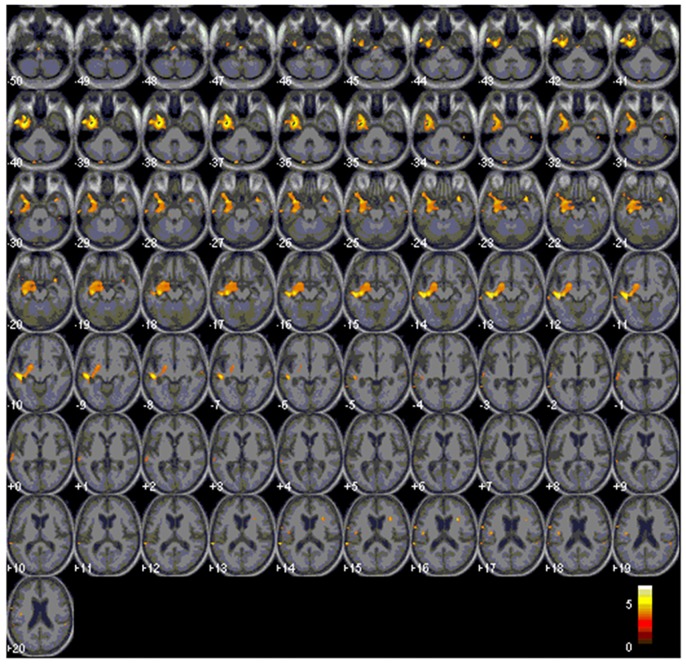
**Red clusters overlaid on the Statistical Parametric Mapping template indicates atrophy in the bilateral anterior and left medial temporal regions that correlates with lower scores in the environmental sound picture matching test**.

When the total scores in sound-picture matching were entered as covariates in the statistical model, there was a correlation between low scores and clusters of atrophy along the left medial temporal and bilateral anterior temporal regions (Figure 6; Table 3).

## Discussion

### Major Findings

From the experiment design, the pitch and melody tests are more of a perceptual test, whereas the environmental sound naming and matching test is more of a semantic association test. The results validated the initial hypothesis that right superior temporal and supra-marginal region atrophy are related to poor melody perception, while the left medial temporal and bilateral anterior temporal regions is related to environmental sound perception. In sub-group comparisons, SD is the only group that has impairment in both tasks. These anatomy-driven differences may explain why the SD-R group has significant impairment in both melody and environmental sound tasks.

### Dementia Severity May Interfere with Perceptual Attention

The pitch discrimination test can also be regarded as an attention test rather than a frequency discrimination test due to the wide frequency ranges between each pitch interval in our experiment. As such, the inverse relationship between pitch score and dementia severity suggests that the auditory attention deficits as the dementia progress. Of specific notes, as the dementia subgroups in this study have no difference in CDR scores, the lower scores in SD, especially SD-R, suggest additional pitch perceptual impairment rather than mere inattention to the task. As the case numbers were small, further validation is needed.

### Scale Violate Melody and Anatomy Correlation

To accurately perform on the melody perception test, the participants need not only perceive the individual pitch, but also store the incoming auditory information long enough for later comparisons. This necessary memory retention time is 3.8–6.4 s (mean, 5.1 s; Peretz et al., 2003). Because this very short implicit memory is at the “bottom” of the perceptual-cognitive system, the process by which it influences response to auditory stimuli is referred to as “bottom-up processing” and is based on immediate sensory experiences (Kubovy and Howard, 1976). In such situations, those with impaired pitch perception and those with limited musical echoic memory (Kubovy and Howard, 1976) may experience difficulties in the melody perception task. As the poor melody perception group was intermixed with different dementia diagnosis, our study results suggested the importance of the right supra-marginal and superior temporal regions for the “bottom-up processing” of violated scales in the dementia groups.

One epidemiologic study has suggested that SD-L occurs about three times more frequently than SD-R (Thompson et al., 2003). Some researchers consider that the lower prevalence of SD-R is related to tasks used in reaching a clinical diagnosis because language ability is relatively spared in the early stage of SD-R. From the functional correlation of melody scores with VBM in this study, we consider that the testing of melody perception may increase the sensitivity in detecting degenerative processes with greater right temporal lobe involvement.

### Environmental Sound Perception Related to Semantic Knowledge

SD is the only group that showed impairment in environmental sound tests. The cognitive process in the environmental sound test requires higher-level processing and interaction with the long-term and semantic memory systems (i.e., “top-down processing”). Although this schema-driven grouping is not possible without prior primitive grouping, the environmental sound stimuli used here are simple so that the effect of bottom-up processing may be ignored. Clinical studies have revealed that word-finding and comprehension difficulties in SD patients are related to semantic deficits (Hodges, 2001; Hodges and Miller, 2001; Nyatsanza et al., 2003). It can be speculated that impaired environmental sounds task in SD may also be related to the inability to associate already learned sounds or pictures. A lack of correlation between environmental sound score and melody scores in our study also suggests that these two non-verbal modalities are mediated by different cortical systems. Our VBM study suggested that environmental association is more lateralized to the left hemisphere and anterior temporal regions, whereas melody perception is more on the right temporal-parietal regions.

### No Category Specificity in the Environmental Perceptual Test

Neuro-psychological studies have shown that categorizing items is a basic operation of the semantic system (Gainotti, 2000). Various theoretical models have been proposed to explain the cognitive mechanism (Caramazza and Shelton, 1998; Ishai et al., 2000) but the anatomical organization of category-specific semantic information is relatively controversial. The posterior visual-association cortices (Mummery et al., 1998; Perani et al., 1999), anterior temporal lobes (Gainotti, 2000, 2015), left dorso-lateral peri-Sylvian regions, and left inferior frontal cortex (Chao et al., 1999; Devlin et al., 2002) have all been reported. However, the environmental sound study results here have not detected differences among four categories in the dementia groups, although the patients have major temporal or frontal lobe involvements.

Meanwhile, the environmental perceptual test with related anatomical correlates in this study was performed by the correlation analysis. Therefore, the finding emphasized on the left temporal structure was not driven by the patients of SD-L. According to the function MRI data on audiovisual object processing, the non-verbal audiovisual matching tasks were related to increased activation in the right fusiform area (Hocking and Price, 2009) which was not different from our result. Possible explanations for the discrepancy may be related to the shared left-lateralized network in both the verbal and nonverbal domains in conceptual processing (Thierry et al., 2003; Thierry and Price, 2006). The correct audio-visual matching of the environmental sound-picture task here required intactness for categorization, comprehension and selection with conceptions of objects. As these patients with dementia may show impairment in some of these tasks, the differences of degenerative processes may have driven part of the difference in the audio-visual networks.

Another possible explanation may be related to the compensatory mechanisms that serve as a protective buffer in the degenerative processes. The brain reserve implies differences in the quantity of available neural substrate (Freret et al., 2015) that reduces the pathological impacts (Chang et al., 2016b). Our correlation model could only point out the atrophic regions that were related to the impairment of task scores. The compensatory mechanism that also plays a role could only be delineated by the functional neuroimaging approach which is worth noting.

### AD and bv-FTD Were not Different from Control in Nonverbal Sound Perception

Both the AD and bv-FTD groups have no differences with the controls in all three tasks, although they also have temporal region involvements (Rosen et al., 2002a,b). From our VBM results, both AD and bv-FTD showed relatively sparing on the anterior temporal and right superior temporal regions as compared with SD. Therefore, the ability of melody and environmental sound perception may not be affected. Other possible mechanisms include paradoxical functional facilitation from other spared Brain region (BAs; Miller et al., 2000) or relatively good integrity of the right hemisphere (Polk and Kertesz, 1993) in retaining musical abilities.

### Limitation

There are several limitations to this study. First, we selected patients with mild to moderate stage dementia and each consisted of small sample sizes. The study design was to delineate the diagnostic differences. The small sample size in each diagnosis might limit the theory to a selected clinical dementia stage. Second, the conclusions with regards to the upstream and downstream relationships of auditory processing pathways were based on the literature review (Kubovy and Howard, 1976). This does not imply that all of these factors (i.e., musical echoic memory, melody task scores and pitch perceptions) are inter-related. The effect of melody task scores may be entirely independent of the effect of pitch perception or musical echoic memory. Finally, although we carefully select patients with similar age ranges, the role of aging process on nonverbal perception ability (Deal et al., 2016) may be highly variable that could form some of the discrepancies from the functional data tested in healthy younger subjects (Hocking and Price, 2009).

## Conclusion

In conclusion, the anatomy of melody perception and semantic association of environmental sound explored in dementia patients show diverse cortical atrophy due to neuro-degenerative disease. This study provides lesion data suggesting that right supra-marginal and superior temporal atrophy are associated with poor melody perceptual ability. Atrophy of the left medial temporal and bilateral anterior temporal regions is linked to the impaired association of environmental sounds with words or pictures. These findings not only complement existing data on the effect of lesions, but also suggest that non-verbal tests may significantly facilitate the investigation of hemisphere laterality in the clinical setting.

## Author Contributions

P-HL participated in the design of the study, drafted the manuscript and performed the statistical analysis. H-HC, N-CC, W-NC, C-WH, Y-TC, S-WH and C-WH participated in the sequence alignment, clinical evaluation of patients and helped draft the manuscript. C-CC helped to draft the work and revise it critically for important intellectual content. All authors read and approved the final manuscript.

## Funding and Acknowledgments

This work was supported in part by grants from Chang Gung Memorial Hospital (CMRPG860171, CMRPG8D0771, CMRPG8E0541), and Ministry of Science and Technology (104-2314-B-182A-026 -MY2).

## Conflict of Interest Statement

The authors declare that the research was conducted in the absence of any commercial or financial relationships that could be construed as a potential conflict of interest.

## Footnotes

^1^http://www.fil.ion.ucl.ac.uk/spm
